# Editorial: Human-Centric Security and Privacy

**DOI:** 10.3389/fdata.2022.848058

**Published:** 2022-02-17

**Authors:** Surya Nepal, Ryan K. L. Ko, Marthie Grobler, L. Jean Camp

**Affiliations:** ^1^CSIRO's Data61, Eveleigh, NSW, Australia; ^2^Cyber Security Cooperative Research Centre, Joondalup, WA, Australia; ^3^School of Information Technology and Electrical Engineering, The University of Queensland, Brisbane, QLD, Australia; ^4^School of Informatics, Computing and Engineering, Indiana University, Bloomington, IN, United States

**Keywords:** cyber security, human-centric security, usable security, usable privacy, HCI and cyber security

Technological solutions alone cannot solve security and privacy problems in emerging technologies. The involvement of human actors and their dynamic behavior introduce a range of factors that necessitates a deep dive into human-centric security and privacy aspects. The goal of this Research Topic was to explore the role that the human plays within the security and privacy domain, and how the humans should interact with the security and privacy aspects. This Research Topic specifically looked at what human-attributable factors impact or affect the perception and adaption of security and privacy. Traditionally, the human player has often been attributed as the weakest link in the technology system, with prior research looking into traditional aspects of human usage of technology. For instance, users are often hardly able to understand the security and privacy-related messages generated by these systems since they are written by tech-savvy developers. Even computer science graduates have difficulties in understanding some of the security and privacy messages generated by modern computer applications, including mobile phones, web applications, etc. Many recent data breaches are attributed to users, deliberate or accidental. The challenge is how to build the security and privacy solutions usable by end-users.

Toward addressing this challenge, our focus with this Research Topic is to facilitate a paradigm shift on how we address security and privacy problems, moving away from a technology focus to the use of a socio-technological dimension. Including both hypothesis and theory perspectives, as well as original research and user studies, this collection of articles deep dives into the exploration of novel socio-technological solutions developed for usable security and privacy. Traditionally, research in cyber security focuses on the technical specifications of the systems and the usage of these systems. A number of advancements have been made in the development and design of the human-centric cyber security domain, bringing a fresh perspective and new insights to how the user, usage and usability of systems combine to play a role in cyber security. A good overview of the current status of human-centric security and the boundaries of the discipline is presented in Grobler et al. as a detailed *case study in usable security and privacy*. They present a survey of existing literature, and classify developmental efforts through existing research works based on the human centric security design, implementation and deployment of the work according to the user, usage and usability elements.

In addressing the role that human-centric security and privacy play, the research conducted by Rao and Pfeffer investigated *consumers' views on privacy and security*. They studied the types of privacy expectations to enhance our understanding of why humans find security and privacy challenging. By considering expectations-related theory in non-privacy literature, they proposed a conceptual model of privacy expectation with four distinct types – Desired, Predicted, Deserved and Minimum – and validate the model using an empirical within-subjects study. They identified that human factors play a critical role in the privacy expectations that people have and that any studies in this field should specifically consider age as a discerning factor.

The research conducted by Nasser et al. provides insight into *usable security by design*. They investigated the roles that cue utilization and cognitive load play in the recognition of phishing emails in a study that supplements the traditional research on users' susceptibility by rather focusing on decision-making strategies for skilled detection. Their study affirmed that a higher cue utilization was associated with a greater likelihood of detecting phishing emails, although demographical factors were found to not have a significant effect on cue utilization. Interestingly, their study found no significant difference in the types of cues used across cue utilization groups or performance levels, and that variation in cognitive load had no effect on phishing detection, nor was there an interaction between cue utilization and cognitive load.

In a similar vein but focused on *usable privacy by design*, the work conducted by Herbert et al. builds on the modern day norm that digital interactions are common place and that privacy considerations are not front of mind in the global society. Specifically, they investigated the willingness of digital natives to self-disclose private data and information from psychological domains including their person, social and academic life, their mental health as well as their health behavior habits. They further examined to what extent the participants' self-disclosure behavior can be modulated by experimental induction of privacy awareness or trust in privacy. This work extends the previous literature on human-centered privacy and can give important insights into self-disclosure behavior of young people and the privacy paradox.

The study conducted by Reuter et al. investigates this discipline through a usability study focused on three end-to-end encryption technologies for securing e-mail traffic. They specifically looked at PGP, S/MIME, and Pretty Easy Privacy (pEp) and found that users seldom apply existing and available encryption technologies to secure their e-mail communication, despite the availability of these technologies. To a large extent, their study uncovered a general lack of awareness of these technologies—60% of their participants were unaware of the existence of encryption technologies and as a result has not tried to use it before. They further found that users generally found the management of public keys overwhelming and the setup process troublesome. This work clearly demonstrates the close link between *human computer interaction in security and privacy* and its alignment with human-centric security and privacy.

In conclusion, this Research Topic has aimed at establishing a baseline understanding of the newly evolving domain of human-centric security and privacy. This collection of articles elevates the current understanding of cyber security and privacy, with a humanistic lens, to provide a bit of definition and substance to the evolvement of the discipline.

## Author Contributions

All authors listed have made a substantial, direct, and intellectual contribution to the work and approved it for publication.

## Conflict of Interest

The authors declare that the research was conducted in the absence of any commercial or financial relationships that could be construed as a potential conflict of interest.

## Publisher's Note

All claims expressed in this article are solely those of the authors and do not necessarily represent those of their affiliated organizations, or those of the publisher, the editors and the reviewers. Any product that may be evaluated in this article, or claim that may be made by its manufacturer, is not guaranteed or endorsed by the publisher.

